# Pituitary carcinoma diagnosed on fine needle aspiration: Report of a case and review of pathogenesis

**DOI:** 10.4103/1742-6413.67108

**Published:** 2010-08-05

**Authors:** Tatiana V. Yakoushina, Ehud Lavi, R. S. Hoda

**Affiliations:** Department of Pathology and Laboratory Medicine, New York Presbyterian Hospital, Weill Cornell Medical College New York, New York

**Keywords:** Fine needle aspiration, pathogenesis, pituitary adenoma, pituitary carcinoma

## Abstract

Pituitary carcinoma (PC) is a very rare entity (0.2% of all pituitary tumors), with only about 140 cases reported in English literature. There are no reliable histological, immunohistochemical or ultrastructural features distinguishing pituitary adenoma (PA) from PC. By definition, a diagnosis of PC is made after a patient with PA develops non-contiguous central nervous system (CNS) or systemic metastases. To date, only three cases of PC have been reportedly diagnosed on fine needle aspiration (FNA). Two of the reported cases were diagnosed on FNA of the cervical lymph nodes and one on FNA of the vertebral bone lesion. Herein, we present a case of PC, diagnosed on FNA of the liver lesion. In this case, we describe cytologic features of PC and compare them to histologic features of the tumor in the pituitary. Clinical behavior of tumor, pathogenesis of metastasis and immunochemical and prognostic markers will also be described.

## INTRODUCTION

The definition of a pituitary carcinoma (PC) includes coexistence of adenohypophyseal tumor and non-contiguous craniospinal or systemic metastases.[1,2] In the absence of metastatic disease, this tumor cannot be termed as a PC, according to the standard World Health Organization (WHO) criteria.[1] Pituitary carcinoma is a very rare entity representing approximately 0.2% of all pituitary tumors.[2,3] There are about 140 cases reported in the English literature for the same.[4,5] The tumor occurs mainly in adults (mean age of 44 years) with equal sex predilection, with a latency period of seven years on average after the initial diagnosis of PA, although this may vary depending on the tumor subtype.[5]

Systemic metastases of PC are usually amenable to fine needle aspiration (FNA) diagnosis. With high sensitivity and specificity, the accuracy of FNA is comparable to that of frozen section. Although cytopathologic features of pituitary adenomas (PAs) are well-described in squash preparations, those of extracranial metastatic PC are not. Only three cases have been diagnosed by FNA.[6,7] We present an additional case of PC with liver metastasis, where we describe cytohistologic features, discuss pathogenesis of metastases and role of immunochemical and prognostic markers.

The majority of tumors in the sella turcica are PAs. They represent 10 – 15% of cranial masses presenting clinically and occur mainly in adults, with equal sex predilection.[1,8] Approximately 50% of PAs can become invasive and infiltrate surrounding structures (dura, bone and brain), while the vast majority of these still remain clinically benign.[5,8,9] The most clinically useful demonstration of invasiveness is sphenoid and cavernous sinus extension on pituitary MRI. Although invasiveness is not indicative of malignancy, it probably puts the patient at risk of developing a PC. Prognosis depends, to a major extent, upon successful resection of the tumor at initial operation.

While most PAs are slow growing and well-demarcated or locally invasive, a minority are more aggressive and/or exhibit morphologic and/or immunohistochemical features of atypical adenoma. Only rare tumors, most presenting as invasive macroadenomas, give rise to distant craniospinal and/or systemic metastases.[4,10]

In our case, a 51 -year-old woman initially underwent transnasal resection for null-cell pituitary macroadenoma. Macroadenoma, by definition, is a pituitary adenoma measuring more than 10 mm in greatest dimension.[1,11] In this case, adenoma was called null-cell because no pituitary hormone production could be demonstrated either clinically or immunohistochemically.[1]

## CASE REPORT

**Figure 1:**
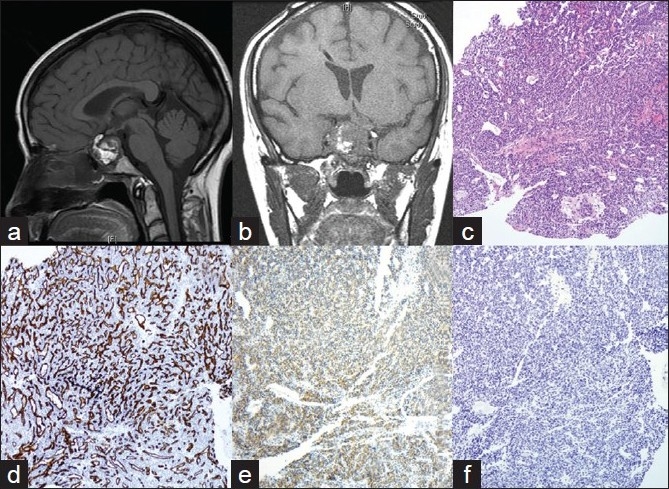
a, b) CT-images of pituitary tumor. Heterogeneous enhancement of pituitary lesion on contrast. a) First recurrence of pituitary tumor; b) Third recurrence of pituitary tumor in the sella turcica; c-f) Histology and immunohistochemistry of the first recurrence of pituitary tumor; c) H and E (10x): Tumor is composed of dense monotonous population of cells; d-f) Immunohistochemical stains; d). CD31 (10x) Tumor displays high vascularity; e) Synaptophysin (10x). Tumor shows diffuse cytoplasmic positivity for Synaptophysin. f) Prolactin (10x). All pituitary hormones, including Prolactin, are negative

**Figure 2:**
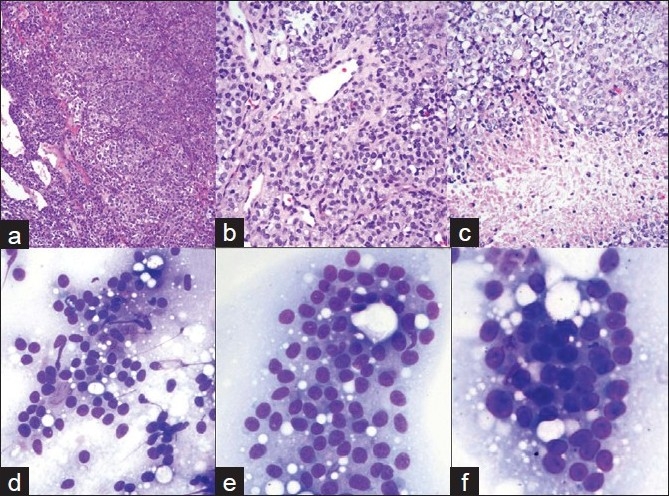
a-c). Histology of recurrent pituitary neoplasm. Dense, highly cellular sheets and nests of monotonous cells with ovoid nuclei and focal clear cell features. Tumor is highly vascular. Areas of necrosis; d-f) Cytology of pituitary carcinoma. FNA of the liver lesion. d, e) 20x; F. 40x: Loose clusters and microacini of monotonous, moderate size cells with eccentrically located, round to oval, mildly pleomorphic nuclei with coarsely granular chromatin and small nucleoli. Cytoplasm is ill-defined due to its fragility

**Figure 3:**
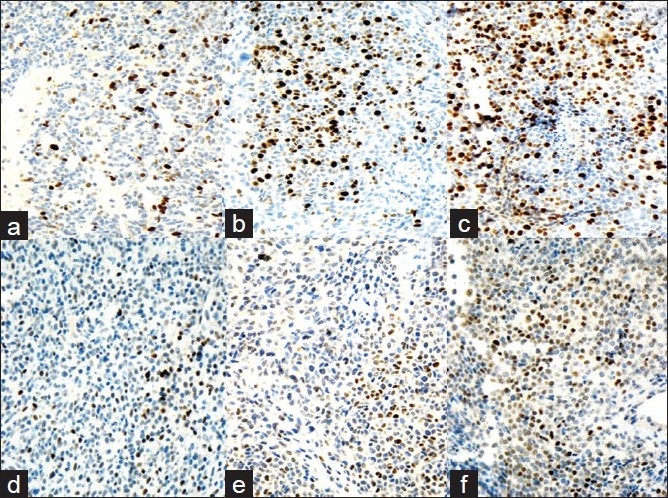
Ki-67 LI immunoreactivity progression. a) First recurrence: Ki-67 LI <3%; b) Second recurrence: Ki-67 LI is ~3-10%; c) Third recurrence: Ki-67 LI >10%. d-f) p53 immunoreactivity progression. a) First recurrence: rare nuclear positivity for p53. b) Second recurrence: focal accumulation of p53-positive cells. c)Third recurrence: >50% of the cells in PC show nuclear positivity for p53

A 51 -year-old woman, married with two children, smoker, presented with visual impairment and headache in November 2005. She recalls remote history of amenorrhea with galactorrhea from age 26 to age 36. MRI showed a pituitary macroadenoma. She underwent transnasal trassphenoidal resection at another institution. Because her tumor had extended into the cavernous sinus, it was not removed completely. Initial pathological diagnosis revealed the disease as PA. Two months later, she presented at our institution with recurrent symptoms. MRI revealed a 4.0 cm recurrent pituitary lesion [Figure 1a], extending into the cavernous sinus, which was resected. Histology showed dense, highly cellular sheets and nests of monotonous cells with ovoid nuclei and focal clear-cell features [Figures 1c, 2a–b]. The tumor exhibited increased vascularity and was surrounded by fibrosis and fat necrosis, consistent with previous surgery. Immunohistochemical stains for Synaptophysin [Figure 1e] and CD31 [Figure 1d] were positive, and all pituitary hormones, including prolactin (PRL), were negative [Figure 1f]. Ki- 67 labeling index (LI) was <3% [Figure 3a] and *p*53 [Figure 3d] showed rare positivity. Pathologic diagnosis at this time was null-cell PA. Small fraction of the tumor extending into cavernous sinus was left behind. Therefore, the patient underwent six months of radiation therapy.

Two years later, the patient presented with severe headaches. MRI detected obstructing hydrocephalus. Cerebral spinal fluid (CSF) cytology was benign. A ventriculoperitoneal (VP) shunt was placed [Figure 1b] and recurrent tumor was resected. Histology [Figure 2c] revealed sheets of neuroendocrine cells with bland nuclei, moderate cytoplasm and focal clear-cell features infiltrating fibrous tissue. The specimen also contained areas of fibrosis, fibroblasts, blood vessels, hemosiderin and focal necrosis, consistent with infarction or treatment effect. The tumor was morphologically and immunohistochemically similar to the previous resection. Ki- 67 LI was in the range of 3 – 10% [Figure 3b]. *p*53 showed focal positivity [Figure 3e]. A diagnosis of recurrent null-cell adenoma was rendered. Six months later, in November 2008, the patient presented with a symptomatic third recurrence and underwent a fourth resection.

Histology was similar to the prior resection. Ki- 67 LI at this time was >10% [Figure 3c], *p*53 showed positivity in more than 50% of the cells [Figure 3f]. Pathologic diagnosis was consistent with recurrent invasive pituitary neoplasm with a note that if metastatic dissemination of this tumor was evident, a diagnosis of PC should be considered. Two weeks later, abdominal ultrasound demonstrated multiple liver lesions. The patient was also found to have multiple lung and humoral bone lesions, and abdominal lymphadenopathy. FNA was performed on a 2.0 cm liver lesion using a 22 - gauge needle. Air-dried Diff-Quik stained slides were prepared. Smears showed loose clusters and microacini of monotonous moderate sized cells with mildly pleomorphic, round to oval eccentrically-placed nuclei with coarsely granular chromatin and small nucleoli. The cytoplasm was moderate in amount, clear and ill-defined or absent due to its extreme fragility [Figures 2d, e, f]. Touch imprints from concurrent core biopsy (CBx) of the liver mass showed uniform solid sheets of tumor cells. Overall, morphology was similar to the cells observed on FNA. Necrosis and mitotic activity were not observed. Immunohistochemical stains performed on CBx were positive for synaptophysin, CD31, *p*53 (majority of cells) and, Ki- 67 LI was >10%. Immunohistochemical stains for other markers (Hepar, CK7, CK20, RCC, TTF - 1) were negative. Final cytologic and CBx diagnosis was PC-rendered after review of prior pituitary tumor and immunohistochemical stains. The patient underwent whole body Octreotide scan in an attempt to define the usefulness of somatostatin as a treatment modality. Because Octreotide is a somatostatin analog, Octreotide scan is used to select tumors responsive to inhibitory action of somatostatin (somatostatin is an inhibitory hormone secreted by hypothalamus). Somatostatin analogs are currently used in the treatment of GH- secreting and null-cell PAs and PCs. In our case, Octreotide scan was positive in liver and bone lesions and negative in lung lesions. Therefore, somatostatin analogs were not considered helpful in this case. Subsequently, our patient underwent chemotherapy with Temodar, with a good response.

## DISCUSSION

In our case, PA was incompletely resected due to extension into the cavernous sinus, followed by radiation therapy. Patient had three symptomatic recurrences and eventually presented with systemic metastases. She recalled a remote history of amenorrhea with galactorrhea from age 26 to 36 years. The latter could have been due to PRL-secreting PA, or “stalk-section effect (due to compression of the stalk between hypothalamus and pituitary and release of inhibitory action of Dopamin on PRL).[1]

Immunohistochemical stains for Ki- 67 and *p*53 performed on her recurrent tumors showed progressive increase in Ki- 67 expression, as well as some increase in *p*53 positivity [Figure 3].

According to the current approach to PC diagnosis, no reliable markers exist to differentiate between PA and PC. Although some authors emphasize the trend towards more aggressive behavior for lesions with higher Ki- 67 LI (>3%) with or without increased *p*53,[1,4,5,12] the overlap in Ki- 67 LI and *p*53 positivity between benign and malignant lesions is considerable.[1,9,11,13] If even weak immunoreactivity is accepted, then *p*53 staining is observed in majority of unselected PAs. However, prediction of aggressive behavior is usually associated with strong nuclear reactivity. One report describes nuclear and cytoplasmic *p*53 staining in both recurrent and metastatic lesions, but not in primary tumor. It was suggested that there are two groups of PCs which differ in aggressiveness, one with and the other without high proliferative activity and *p*53 expression.[9] Overall, PC discrepancy between histologic appearance and phenotypic behavior remains poorly understood.[3]

Histological and cytologic appearance of PC may vary from bland to distinctly malignant. Mitotic rate can be low or absent.[9,13] Some authors attribute it to delayed tissue fixation or post-treatment phenomena.[9,14] This variation may also be due to the fact that PCs are heterogeneous with respect to proliferative activity. Although assessment of proliferation may be helpful in arousing suspicion as to subsequent tumor invasiveness and/or malignant potential, growth rate is probably not the only major determinant of tumor behavior and carcinogenesis.[1,2,4,14,15]

Significant similarities also exist in the ultrastructural appearance of PC and their benign counterparts such as PA and atypical PA.[4,10] It was noted that invasion, necrosis, nuclear and cellular pleomorphism have no correlation with behavior.[9] Despite the benign histology of many of these tumors, prognosis is poor,[1,3,16] as 66% of patients died due to this disease within one year of diagnosis.[1,4] Although patients with long-term survival have been described, no recognizable factors of increased survival have been found.[2]

Because distinction between PA and PC cannot be made on the basis of histologic or ultrastructural features alone, a new category of “atypical pituitary adenoma” was included in the new 2000 WHO Classification of Endocrine Tumors.[10] It was recommended that invasive PAs with Ki- 67 LI >3% and high *p*53, or patients with Ki- 67 LI >10% regardless of *p*53 be better defined as “atypical PAs” and these patients be closely followed with serial MRIs.[1,5] Shibuia et al.[4] in a study on 65 pituitary tumors, demonstrated Ki- 67 LI of 0.8% in primary PA, 3.6% in recurrent PA, 1.7 – 4.6% in invasive PAs and 7.8 – 12% in PCs. According to the other study,[4] *p*53 overexpression was detected in 0% of PAs, 15% of invasive PAs and ~100% of PCs. Mutations of *p*53 tumor suppressor gene have been reported in a small subset of PCs and are usually associated with a high percentage of tumor cells overexpressing the p53 protein. It suggests that in the majority of PCs the protein identified is wild type[17]. The majority of the studies noticed that *p*53 expression correlated with the proliferative state of the tumors, as may be assessed by the Ki- 67 LI.[11] Although histologically “atypical PAs” do not necessarily behave clinically as “atypical”, a high index of suspicion for these lesions is currently recommended.[1,3,8,15] It was also noted that there does not seem to be a significant correlation between Ki- 67 LI and tumor size of PA, even if this index is considered as a useful marker in the determination of the infiltrative behavior of these tumors.[11] Clinically, the name “atypical pituitary adenoma” is used to describe tumors that recur or progress despite resection and radiotherapy. These tumors often do not appear overtly malignant by histological criteria, but exhibit aggressive phenotypic behavior.[3]

Regarding other helpful markers, some authors report increased vascularity in more aggressive pituitary tumors.[1,4,5] Some studies have indicated that genetic defects, including ones involving oncogenes and tumor suppressor genes, play a role in pituitary tumorigenesis and progression.[1,10] p27, a cell cycle inhibitor, is decreased in PC compared to PA and normal adenohypophyseal cells.[1,5,14] According to some authors, a mutated *H*-ras oncogene has been observed in metastases from PC but not in primary adenoma.[2,4] Also high levels of *c*-myc oncogene have been found in a variety of aggressive pituitary tumors.[5] Increased proliferating cell nuclear antigen (PCNA) index and her2/neu membrane staining were demonstrated in primary PC and its metastases.[2,4,5,18] One study showed that multiple endocrine neoplasm type I gene, *menin*, was significantly decreased in PA and was undetectable in PC.[5] Although DNA ploidy status (diploid *vs*. aneuploid) has been shown to be a useful prognostic indicator in a number of human neoplasms, two recent studies showed this not to be true of PC.[8,14] Although microvascular density is usually higher in PCs, there is no clear distinction between these tumors and benign or invasive PAs on the bases of CD31 staining, and no correlation of CD31 positivity with Ki- 67 LI exists.[4,5] However, it has been shown that the majority of PCs exhibit increased matrix metalloproteinase activity that is related to extracellular matrix degradation promoting angiogenesis and tumor invasion.[1,8]

As is true of PAs, only limited conclusions could be drawn regarding the secretory activity of PCs on the basis of their morphology. Most (75 - 88%) of the PCs are functional, with PRL-producing (33%) PCs and ACTH-producing (42%) PCs being more common.[1,2,4] Null-cell PCs represent 12% of the reported cases. Before the 1970s, PRL levels were not measured and immunohistochemical staining was not performed. Therefore, a significant number of PRL-producing PCs were probably unrecognized, making data from older literature somewhat unreliable.[4]

PCs with metastases outside the CNS are more common than craniospinal metastases, with the liver being the most common site of metastatic spread. Other metastatic sites include bone, lung, and lymph nodes in descending order of frequency.[15]

Reportedly, the interval between diagnosis of pituitary neoplasm and development of metastases ranges between 4 months and 30 years (average 8 years). It is shorter for PRL-secreting adenomas, which tend to produce systemic metastases more often, whereas ACTH-secreting PCs more commonly produce CNS metastases.

There is no standard treatment for PCs.[15] Treatment modalities include surgery, radiation for incompletely resected tumors, and chemotherapy. The latter also includes hormonal therapy with dopamin agonists for PRL-secreting tumors and somatostatin agonists for GH-secreting and null cell tumors.[2,4]

Histological differential diagnosis of initial pituitary tumor in our case included null-cell pituitary adenoma, hemangiopericytoma, lymphoma, other tumors of the sellar region and metastases from breast and lung among other possibilities.[4,5,7,19] Cytologic and histologic differential diagnosis of systemic metastasis included primary or metastatic neuroendocrine tumors, especially carcinoids, lymphoma, multiple myeloma, metastatic acinar cell carcinoma of the pancreas, metastatic renal cell carcinoma and metastatic medullary cell carcinoma.[6,7] The distinction between a plasmacytoma/multiple myeloma and PC can be difficult as the cytological findings of the two neoplasms are quite similar. Initial diagnosis of lymphoma on FNA of PC can be entertained by the cytopathologist because the tumor often consists of a noncohesive, monomorphic population of cells with plasmacytoid features.[7] Distinction between pituitary carcinoma and metastatic carcinoids may be impossible in the absence of detailed clinical data.[6] Cytohistologic and immunohistochemical features are very similar in both tumors. In our case, recognition of a history of recurrent null-cell pituitary adenoma aided us with the diagnosis of PC. Cytologic features of liver metastases of renal cell carcinoma and thyroid medullary carcinoma can be confused with those of pituitary carcinoma. Clinical, radiological features (no tumor mass in the kidney or thyroid gland) and immunohistochemical studies can reliably differentiate between these entities.

Cartwright *et al*.[7] first described cytopathologic features of PC in two cases of PCs with cervical lymph node metastases. Out of which, one was a case of 28 -year-old woman with GH-producing PC with cervical lymph node metastasis. FNA from a cervical lymph node of this case revealed features similar to our case. The second case in their report occurred in a 46 -year-old woman with non-secreting PC with cervical lymph node and paraspinal metastases. FNA of the cervical lymph node showed loosely cohesive tumor clusters with significant nuclear pleomorphism and peculiar cytoplasmic vacuoles. Prominent nucleoli were observed in many of the tumor cells, and mitotic figures were prominent. Koray Ceyhan *et al*.[6] described cytological features of cervical vertebral bone metastases of ACTH-producing PC. Neoplastic cells in this report were also similar to our case.

Pathogenesis of metastatic spread in PC still remains unclear. One of the possible mechanisms of spread is a hematogeneous pathway, through rich anterior pituitary portal system into cavernous and petrosal sinuses, followed by jugular vein and with venous return to the lungs.[5] Nevertheless, pulmonary sites for the metastases of PCs are rarely seen.[4] For reasons still unclear, liver metastases of PC are most common.[15] Hematogenous dissemination has been reported only in a few cases, primarily of ACTH-producing carcinomas.[12] Our case is one of the rarest cases of hematogenously disseminated null-cell PC. Another theory of PC pathogenesis postulates lymphatic invasion within the skull base and soft tissue, which may lead to cervical lymph nodes metastases. Also, there is a reported connection between intracranial perineural spaces and lymphatic plexus in the neck. This mechanism can explain ipsilateral cervical lymph nodes metastases in some intracranial tumors.[5,13] There is an unproved theory about iatrogenic dissemination of pituitary carcinoma. The disruption of vascular barriers during surgical intervention, negative pressure in the intraoperatively opened veins may potentially aspirate tumor cells, similar to the systemic spread of gliomas. Also, fragile newly formed postoperative vessels may become easily penetrated by tumors cells.[2,4,13] Previous radiotherapy has also been implicated as risk factor.[2,13]

Another possible route of spread of PC is along pre-existing cavities. Tumor cells may spread into deep brain structures along Wirchow-Robin spaces (perivascular CNS spaces) creating non-contiguous metastases. They can also spread together with CSF,[5,13,20] resulting in superficial (for ex., subpial and periventricular) metastases along neuraxis. Another possible, although very rare, route of metastatic spread is through the VP shunt.[13] Less than 100 of VP shunt-related metastases have been reported in the English literature.[21] According to some studies, it occurs in <1.0% of patients with obstructive hydrocephalus due to brain tumor.[22] The majority of these patients are children; the most common culprits are germinomas and medulloblastomas. VP-shunt related metastases are mostly located on omental and/or peritoneal surfaces and often in close proximity to the tip of the catheter.[13] The interval between shunt placement and development of metastases ranges between 1 month and 6 years. Up until now, this method of spread has never been reported in patients with PC. Our patient had a VP shunt placed six months before appearance of her metastases.

Two possible mechanisms of PC development were proposed. One, favored by most authors, is adenoma-to-carcinoma sequence.[1,4,5,10] This theory was based on similar histological and molecular markers between primary and metastatic tumors according to some studies.[4] Less commonly, PCs can exhibit an early malignant behavior. They are likely to arise *de novo* from adenohypophyseal cells. This theory developed after some genetic studies demonstrated that primary and metastatic tumors were two individual clones indicating either a *de novo* pituitary tumorigenesis or clonal expansion from the original tumor.[2,4–6] Some authors noticed that clinical development of pituitary insufficiency within a time frame of several weeks is consistent with the development of PC.[16] The most common clinical presentation of PC, according to the literature, is early recurrence after initial pituitary surgery, followed by repeated operations for rapid local growth and tumor extension.[5] Other studies emphasized that patients with incomplete removal of PA due to extension of the tumor into vital structures (cavernous sinus, sphenoid sinus) as well as those undergoing multiple surgeries and/or radiation treatment, are at higher risk of progression of their PA to PC and need constant surveillance, regardless of the time of follow-up.[5]

Clearly, arriving at a workable, clinically meaningful classification of adenohypophyseal tumors that reflect their biologic behavior will require the assessment of cytomorphological and immunohistochemical features, as well as other parameters, such as cell cycle inhibitors, growth factors, growth inhibitors, tumor suppressor genes, and gene mutations.[10]

## CONCLUSION

PCs with extracranial metastases demonstrate typical neuroendocrine features on FNA. In the differential diagnosis, metastatic neuroendocrine carcinomas should be kept in mind. Although some cytomorphologic differences are noticed, in the absence of sufficient clinical data, these two entities might be very difficult to distinguish correctly based on cytologic features alone.

Our case is in agreement with the previously reported conclusion that among the currently available predictive factors for the development of PC, tumor invasiveness is important.[5,6] Whereas, the Ki- 67 LI and p53 protein expression can be very helpful, other immunohistochemical or histological characteristics of PA proved to be of little value in our case. Future studies will focus on identifying those PAs most likely to metastasize and treating them aggressively, before they progress to pituitary carcinoma.[5]

## COMPETING INTEREST STATEMENT BY ALL AUTHORS:

Competing interests are not present in this case.

## AUTHORSHIP STATEMENT BY ALL AUTHORS:

Each author acknowledges that this final version was read and approved.

## ETHICS STATEMENT BY ALL AUTHORS:

As this is case report without identifiers, our institution does not require approval from Institutional Review Board (IRB) (or its equivalent)

## EDITORIAL / PEER-REVIEW STATEMENT:

To ensure integrity and highest quality of CytoJournal publications, the review process of this manuscript was conducted under a double blind model(authors are blinded for reviewers and reviewers are blinded for authors) through automatic online system.
